# Integrase Interactor 1 (INI1) Deficiency in a Lung Cancer Patient Presents Nonresponse to Immunotherapy and Tazemetostat: A Case Report

**DOI:** 10.7759/cureus.42934

**Published:** 2023-08-04

**Authors:** Xiuxiu Chen, Jiaji Wu, Guanchao Pang, Shumei Wei, Pingli Wang

**Affiliations:** 1 Department of Respiratory and Critical Care Medicine, The Second Affiliated Hospital of Zhejiang University School of Medicine, Hangzhou, CHN; 2 Department of Pathology, The Second Affiliated Hospital of Zhejiang University School of Medicine, Hangzhou, CHN

**Keywords:** tazemetostat, immunotherapy, lung cancer, smarcb1, ini1

## Abstract

Integrase interactor 1 (INI1)-deficient lung cancer is extremely rare, often with poor prognosis, and lacks effective treatment. Previous studies have reported the efficacy of immunotherapy and enhancer of the zeste homolog 2 (EZH2) inhibitor tazemetostat in various types of INI1-deficient tumors, such as sarcomas. However, the effectiveness of these treatments in INI1-deficient lung cancer has not yet been verified. We hereby report a case of a patient who was diagnosed with advanced squamous lung cancer with INI1 deficiency and received chemotherapy, immunotherapy, and tazemetostat treatments successively. The patient showed optimal response in the initial chemotherapy combined with anti-programmed cell death protein 1 (PD-1) immunotherapy, made rapid progress in the subsequent stage of maintenance immunotherapy, and showed nonresponse to tazemetostat. To the best of our knowledge, this is the first case of a lung cancer patient with INI1 deficiency who received tazemetostat treatment.

## Introduction

Integrase interactor 1 (INI1/*SMARCB1*) is involved in the formation of SWItch/Sucrose Non-Fermentable (SWI/SNF), which is in charge of global chromatin remodeling. It acts in multiple pathways, including regulating p16 and inhibiting MYC target activation, thus making it a powerful tumor suppressor [1]. INI1 deficiency is most often seen in malignant rhabdoid tumor (MRT), epithelioid sarcoma, and pancreatic undifferentiated rhabdoid carcinoma but is rarely found in lung cancer [2]. Patients with tumors characterized by INI1 deficiency usually have a short survival [3], reflecting the aggressive nature of these tumors.

Recently, *SMARCB1*-negative pediatric MRT and renal medullary carcinoma (RMC) have been proven to be immunogenic, and the effectiveness of immune checkpoint inhibitors (ICIs) has been validated in *SMARCB1*-negative sarcomas [4]. Meanwhile, the loss of INI1 in tumors causes the abnormal enhancer of zeste homolog 2 (EZH2) activity, which can be a target for tazemetostat, an EZH2 inhibitor [5]. A growing number of clinical studies and case reports illustrate the effectiveness of tazemetostat in various types of INI1-deficient tumors. In a limited number of cases, ICI has been used for the treatment of INI1-deficient lung cancer, with poor efficiency observed [6]. However, tazemetostat has never been used for lung cancer with INI1 deficiency. Here, we report a case of a patient who was diagnosed with advanced squamous cell lung cancer with INI1 deficiency and received chemotherapy, immunotherapy, and tazemetostats successively but showed nonresponse to pembrolizumab and tazemetostat.

## Case presentation

**Figure 1:**
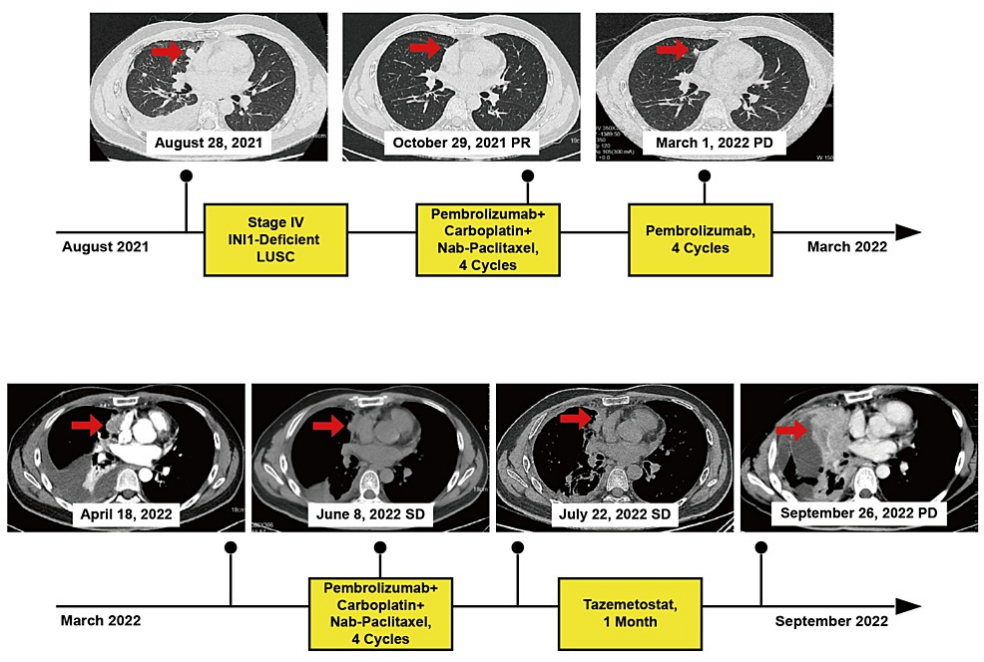
Schematic diagram of the course of treatment and imaging changes in pulmonary lesions Red arrows indicate the target lesions. LUSC: lung squamous carcinoma; PR: partial response; PD: progressive disease; SD: stable disease

A 33-year-old male was admitted to the hospital with right shoulder and back pain on August 28, 2021. He denied any smoking history and any remarkable medical history. Chest computed tomography (CT) scans showed a mass in the middle lobe of his right lung with multiple metastases in the right lung, pleura, and interlobular fissure, and the right pleural effusion was also found (Figure 1). No metastatic lesions were detected except in the right lung and pleura. Lung biopsy of the primary lesion revealed a poorly differentiated squamous cell carcinoma. The immunohistochemistry outcomes were as follows: thyroid transcription factor 1 (TTF-1) scattered +, P40 +, programmed death-ligand 1 (PD-L1) -, synaptophysin (Syn) -, chromogranin A (CgA) -, CD56 -, cytokeratin (CK) (AE1/AE3) +, Ki-67 30%, CK5/6 partial +, estimated glomerular filtration rate (EGFR) +, INI1 -. Therefore, this patient was diagnosed as stage IV squamous cell carcinoma of the right lung (cT2aN0M1a). The Eastern Cooperative Oncology Group (ECOG) score was 1. Since September 2021, he received the treatment of programmed cell death protein 1 (PD-1) antibody (pembrolizumab) combined with chemotherapy of carboplatin and nab-paclitaxel. After three cycles of treatment, the patient’s back pain symptoms disappeared. The chest CT scan showed that the right lung mass became smaller obviously, and the metastatic lesions and right pleural effusion almost vanished. The best efficacy was estimated to be partial response (PR) in accordance with Response Evaluation Criteria in Solid Tumors (RECIST) version 1.1. Then, after completing a total of four cycles of pembrolizumab combined with chemotherapy, he received pembrolizumab maintenance therapy every three weeks.

**Figure 2:**
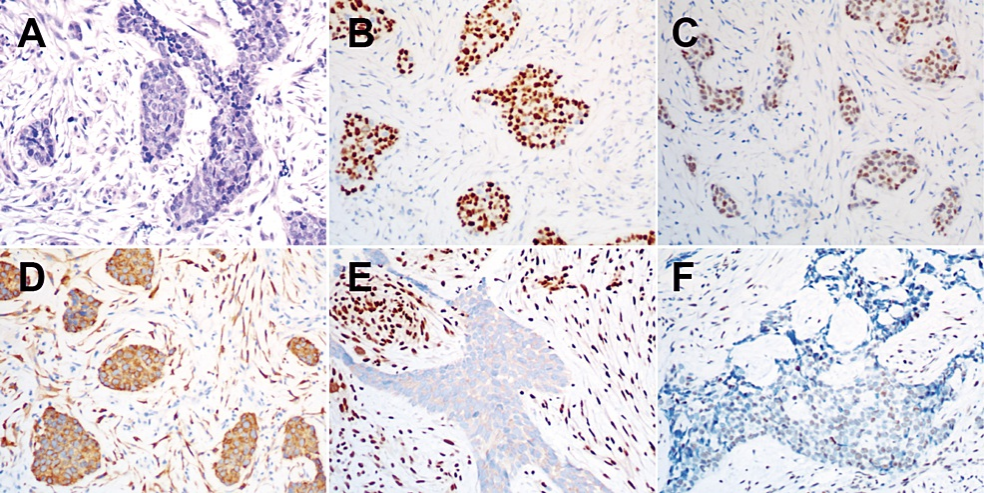
Tumor morphology and immunophenotype (A) hematoxylin and eosin (H&E); (B) P40; (C) thyroid transcription factor 1 (TTF1); (D) cytokeratin (CK); (E) integrase interactor 1 (INI1); (F) Brahma‐related gene 1 (BRG1).

On March 1, 2022, following two cycles of single-agent immunotherapy, the lung CT revealed that the primary and metastatic lesions in the right lung became larger, which were consistent with a progressive disease (PD) (Figure 1). Despite this, the patient remained asymptomatic, so pembrolizumab was continued. On April 18, 2022, the patient presented with cough and dyspnea. CT scan showed a notable progression of the lesions and pleural effusion on the right side. We performed a biopsy on the right pleural nodules, and the results indicate dedifferentiated squamous cell carcinoma with INI1 deficiency. On immunohistochemistry, the tumor stained positively for P40 (mostly +), TTF-1 (mostly +), Ki-67 (40%), CK (AE1/AE3) (diffuse +), EGFR (+), and Brahma‐related gene 1 (BRG1) (+) and negatively for CK5/6, Syn, CgA, CD56, PD-L1, CD117, CD5, and INI1 (Figure 2). Molecular sequencing analysis of the biopsy specimens did not detect *SMARCB1* deletion. Based on the patient's positive response to carboplatin, nab-paclitaxel, and pembrolizumab in the past, the same treatment regimen was chosen again. However, after two treatment cycles, the efficacy was only evaluated as a stable disease (SD). In July 2022, after completing a total of four cycles of treatment, the patient's right chest pain worsened and the lung lesions continued to grow, although it is not obvious (SD). Therefore, from August to September 2022, the patient switched to tazemetostat treatment. However, his right chest pain did not improve, and the CT scan showed the lesions and pleural effusion kept growing, which was evaluated as PD (Figure 1). 

The patient was subsequently enrolled in a clinical trial with a regimen of docetaxel associated with PD-1/cytotoxic T-lymphocyte antigen 4 (CTLA-4) bispecific antibody. After two cycles of treatment, the patient withdrew from the clinical trial due to ongoing progression of the lung lesions. At present, the patient's condition is deteriorating, and he is in the terminal stage of receiving best supportive care.

## Discussion

This report contains a case of squamous cell lung cancer harboring INI1 deficiency, which exhibited a favorable response to carboplatin combined with nab-paclitaxel and pembrolizumab, although the response was of short duration. Notably, both pembrolizumab and tazemetostat were ineffective in this case. By immunohistochemistry, the absence of nuclear labeling of INI1 in tumor cells was confirmed. Further evaluation using molecular sequencing analysis did not find corresponding *SMARCB1* deletion, which implies that the loss of INI1 protein may be due to epigenetic regulation rather than mutation.

Mutations in SWI/SNF subunit genes can be found in nearly 20% of lung cancers, with *SMARCA4*, *ARID1A*, *ARID2,* and *SMARCA2* being the most frequently altered genes. *SMARCB1* mutations are relatively uncommon [7]. *SMARCA4*-deficient undifferentiated tumor (SMARCA4-UT), a malignancy being treatment-refractory to cytotoxic chemotherapy, was newly added to the 2021 WHO Classification of lung tumors [8]. However, the features of lung tumors with INI1 (*SMARCB1*) deficiency have not been systematically generalized. In a study, intrathoracic neoplasms with alterations in the SWI/SNF complexes were stratified in morphologic sub-groups: epithelioid, rhabdoid, mixed, and solid. These tumors were recognized as having various histological origins using immunohistochemical and molecular methods. Most of the patients with these tumors had pleural involvement and died of progressive diseases [3]. The patient in this case was diagnosed with advanced non-small cell lung cancer at a young age. While the initial regimen consisting of chemotherapy and immunotherapy achieved favorable effects, the duration of the response was less than six months. During the maintenance immunotherapy phase, the disease progressed rapidly, which indicated a nondurable response to PD-1 antibody in this patient.

Alterations in the SWI/SNF complex contribute to tumor immunogenicity. Endogenous retrovirus (ERV) re-expression and interferon-signaling activation, which are related to rhabdoid tumor (RT) immunogenicity, depend on *SMARCB1* deficiency [9]. *PBRM1* knockdown decreased immunosuppressive cytokines, and *PBRM1* levels were found to be inversely related to CD8 cytotoxic T-cell infiltration [10]. Inactivation of any of the three genes *ARID2*, *PBRM1,* and *BRD7* enhances the tumor-killing effect of cytotoxic T cells [11]. The above-mentioned studies lay the groundwork for the application of ICI therapy in tumors with SWI/SNF abnormalities, but some clinical studies indicate that not all tumors with SWI/SNF mutations benefit from ICI [12,13]. The available reports on ICI for INI1-deficient lung cancer treatment are limited, and they indicate that ICI is ineffective in treating this type of cancer [6]. Our patient with INI1-deficient lung cancer also showed insensitive to immunotherapy too. Probably, immunotherapy does not work for this particular cancer, but this needs to be supported by a larger sample size study.

Apart from immunotherapy, targeted therapy can be another promising therapeutic option for handling tumors with INI1 deletion. INI1 deletion leads to abnormal activities of EZH2, and activated EZH2 catalyzes the trimethylation of histone H3 lysine 27 (H3K27), which inhibits the transcription of target genes, such as anti-oncogene. Tazemetostat is an inhibitor of EZH2 and helps to ameliorate EZH2-dependent carcinogenesis [5]. Currently, tazemetostat is approved by the Food and Drug Administration (FDA) for epithelioid sarcoma and relapsed or refractory follicular lymphoma. Several cases and clinical trials have reported responses to tazemetostat in INI1-deficient pediatric tumors, sinonasal tumors, and *BRCA*-associated protein 1 (BAP1)-inactivated malignant pleural mesothelioma [14-16]. There have been no studies using tazemetostat in the treatment of INI1-deficient lung cancer, and as far as we know, the present case is the first instance. However, unfortunately, the patient did not respond to tazemetostat.

## Conclusions

We provided a case of lung cancer with INI1 deficiency who presented nonresponsive to anti-PD-1 immunotherapy and tazemetostat, although some studies have demonstrated the potential efficacy of these treatments in other kinds of tumors with INI1 deficiency. Lung cancers with INI1 deficiency demonstrate poor prognosis and lack effective treatments. Further exploration is needed to improve the survival of this disease.
